# DNAvi: integration, statistics, and visualization of cell-free DNA fragment traces

**DOI:** 10.1093/bioinformatics/btag041

**Published:** 2026-01-29

**Authors:** Anja Hess, Dominik Seelow, Helene Kretzmer

**Affiliations:** Exploratory Diagnostic Sciences, Center of Genomic Medicine, Berlin Institute of Health at Charité Universitätsmedizin Berlin, 10117 Berlin, Germany; Max Planck Institute for Molecular Genetics, 14195 Berlin, Germany; Department of Biology, Chemistry and Pharmacy, Freie Universität Berlin, 14195 Berlin, Germany; Exploratory Diagnostic Sciences, Center of Genomic Medicine, Berlin Institute of Health at Charité Universitätsmedizin Berlin, 10117 Berlin, Germany; Institute of Medical Genetics and Human Genetics, Charité Universitätsmedizin Berlin, Corporate Member of Freie Universität Berlin and Humboldt-Universität zu Berlin, 13353 Berlin, Germany; Max Planck Institute for Molecular Genetics, 14195 Berlin, Germany; Hasso Plattner Institute for Digital Engineering, Digital Engineering Faculty, University of Potsdam, 14482 Potsdam, Germany

## Abstract

**Summary:**

DNAvi is a Python-based tool for rapid grouped analysis and visualization of cell-free DNA fragment size profiles directly from electrophoresis data, overcoming the need for sequencing in basic fragmentomic screenings. It enables normalization, statistical comparison, and publication-ready plotting of multiple samples, supporting quality control and exploratory fragmentomics in clinical and research workflows.

**Availability and implementation:**

DNAvi is implemented in Python and freely available on GitHub at https://github.com/anjahess/DNAvi under a GNU General Public License v3.0, along with source code, documentation, and examples. An archived version is available under https://doi.org/10.5281/zenodo.18401705.

## 1 Introduction

Cell-free DNA (cfDNA) is short, nucleosomal-sized DNA released into the extracellular space as cells undergo apoptosis (Lo *et al.* 2021). The use of blood-derived cfDNA originating from tumor cells has gained attention as a potential noninvasive cancer diagnostic tool in the context of liquid biopsies (Cohen *et al.* 2018, Cristiano *et al.* 2019, Fedyuk *et al.* 2023), and is being investigated in large clinical trials across countries with thousands of participants (Klein *et al.* 2021, Nicholson *et al.* 2023, Schrag *et al.* 2023, Jager *et al.* 2025). Interestingly, the size of cfDNA fragments itself can have diagnostic value, as fragment size or fragment size variability alone can discriminate tumor and healthy controls in a wide range of solid tumor entities, including lung, liver, and colon cancer, advancing the field of fragmentomics (Jiang *et al.* 2015, Cristiano *et al.* 2019, Chabon *et al.* 2020, Esfahani *et al.* 2022, Zhou *et al.* 2024, Bao *et al.* 2025, Malki *et al.* 2025).

The first step in analyzing cfDNA samples from liquid biopsies is to visualize, quantify, and size-profile the cfDNA following the principles of gel electrophoresis (Voytas 2001, Green and Sambrook 2019). Typically, this quantification and size determination is performed using automated gel electrophoresis devices, such as the Bioanalyzer or TapeStation (Agilent), which eliminates potential biases introduced by manually prepared gels and enables highly parallelized profiling. Such fragment size evaluations are also an indispensable quality control step prior to high-throughput sequencing.

cfDNA fragmentomics is of interest for its diagnostic potential and therefore requires comparing profiles between different groups (e.g., normal versus tumor). However, free tools that quickly and intuitively integrate, visualize, and compare large sets of electrophoresis-derived cfDNA fragmentation profiles across conditions, prior to expensive sequencing experiments, are currently not available.

Commercial software that accompanies automated electrophoresis devices typically plots only fluorescence intensity values for each sample. This becomes increasingly impractical when more than a handful of samples are visualized and evaluated side by side.

Additionally, these tools typically do not provide statistical metrics critical for evaluating the potential diagnostic implications of fragment profiles, such as per-group differences in nucleosomal fractions and fragment sizes. Integration into automated workflows is not possible, as these programs are designed as graphical user interface (GUI) desktop applications (Application Note. Agilent TapeStation Software 5.1 for the Agilent TapeStation Systems). On the other hand, more elaborate tools and databases that also deliver fragmentomic profiles, such as *FinaleDB* (Zheng *et al.* 2021), *FinaleToolkit* (Li *et al.* 2024), *LBFextract* (Lazzeri *et al.* 2024), or *cfDNAPro* (Wang *et al.* 2025), require sequencing of the DNA, which comes with high costs and long processing times (Chen *et al.* 2022).

Hence, we developed DNAvi as a user-friendly command-line tool for the analysis and publication-ready visualization of cfDNA fragmentomic profiles based on the concept to transform raw DNA electrophoresis images into signal tables (Akbari *et al.* 2010, Park *et al.* 2012, Pavel and Vasile 2012). Other than conventional DNA band callers, DNAvi allows users to provide metadata to group samples into clinically relevant categories, integrate multiple DNA traces into averaged profiles, compute relevant cfDNA-related metrics, and perform statistical testing between groups.

## 2 Implementation

### 2.1 Overview

**Figure 1:**
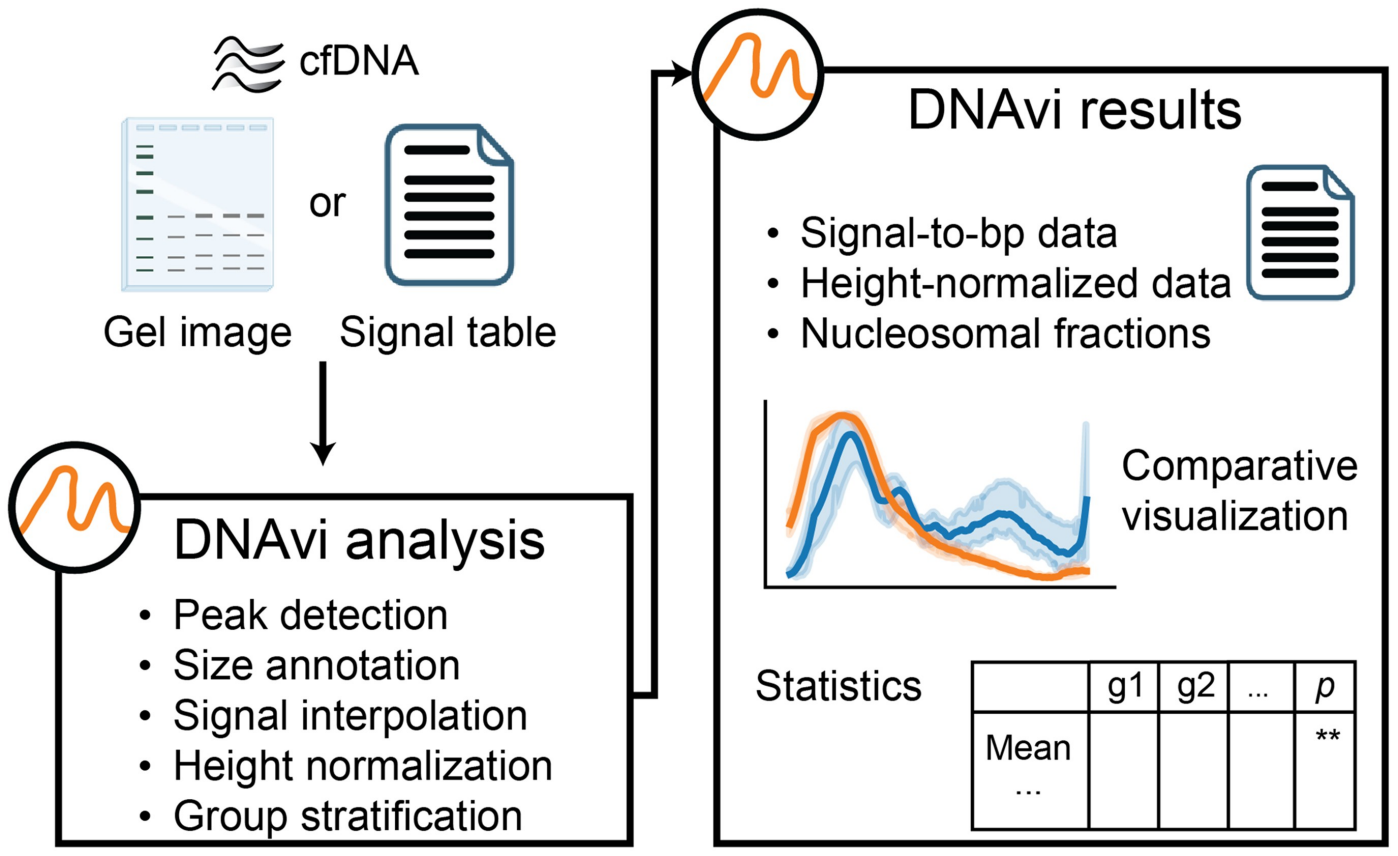
Schematic overview of the DNAvi workflow. Gel images or signal tables from cfDNA gel electrophoresis are analyzed and processed for subsequent group-versus-group visualization and statistical comparisons. Parts of the graphics were created with BioRender.

DNAvi is a command-line tool implemented in Python 3, using standard libraries such as matplotlib (Hunter 2007), seaborn (Waskom 2021), scipy (Virtanen *et al.* 2020), and scikit-image (van der Walt et al. 2014). Its core principle is the transformation of user-supplied raw signal tables or computed images from gel electrophoresis data into signal intensities that represent normalized, concentration-independent fragment length distributions (Fig. 1). These distributions can be visualized, compared, and quantified in an automated, user-friendly manner. Final result tables and plots, as well as intermediate results (e.g., image annotations, image-derived raw signal tables, and peak statistics) generated by DNAvi, are stored locally and readily accessible.

### 2.2 Installation, input, and workflow

DNAvi is a command-line tool and is simply installed through pip. Alternatively, its github repository can be cloned after the required Python libraries have been installed. The tool is started by calling *dnavi* from the command-line, or *DNAvi.py* with Python (version 3.12). DNAvi accepts gel images or cfDNA electrophoresis signal tables as input. The latter may be obtained from automated gel electrophoresis devices, such as the Agilent Bioanalyzer, Agilent TapeStation, and other similar automated gel electrophoresis devices (e.g., the Qsep Fragment Analyzer) (Agilent TapeStation and Bioanalyzer Systems 2024, Nippon Genetics: Qsep Fragment Analyzer 2025). Multiple files can be screened by providing a directory as input. Optionally, a metadata file can categorize samples by attributes such as sex, age, or disease. The signal table input requires a DNA intensity matrix for each sample and a text file to annotate the size standard. Signal tables are widely accessible, since they constitute a standard output of state-of-the-art electrophoresis machines. If not available, DNAvi can also generate signal tables from agarose gel images in JPEG, JPG, or PNG format, following defined quality standards outlined in the online documentation (https://dnavi.readthedocs.io/).

In brief, the DNAvi workflow comprises an analysis, visualization, and statistics module. First, the size standard is used to calibrate size information for all intensity values by detecting peaks and interpolating intermediate fragment sizes. A subsequent normalization step equalizes peak heights, thereby enabling DNA concentration-independent analysis of peaks. These data are then visualized, metrics such as mean, median, and modal fragment size are computed, and undergo statistical testing for differences between groups based on metadata. Simple group-stratified plots are generated to facilitate comparisons between conditions. Finally, the results are stored locally (Supplementary Data, available as supplementary data at *Bioinformatics* online).

### 2.3 Functional modules

#### 2.3.1 DNA image conversion to signal intensity table

Upon gel image input, DNAvi uses standard image processing routines to extract lane-specific signal profiles. First, to determine the individual DNA lanes, the images are converted to grayscale, filtered, and thresholded using Otsu’s method (Otsu 1979). Then, intensity values are extracted along the vertical axis of each lane, with center-aligned pixel columns used to represent band intensities. The resulting signal table is compiled into a dataframe, with each column corresponding to a lane and each row to a gel position. It is then exported in CSV format for downstream analysis.

#### 2.3.2 Intensity table, peak detection, size standard handling, translation with interpolation, and normalization options

Once the signal intensity table has been generated, DNAvi detects and calibrates the size standard (‘ladder’) peaks, which, by default, are expected in the first lane but can be retrieved from alternative lanes if specified by the user. The detected peaks are then matched to the user-supplied base pair sizes from the size standard annotation file. If the number of peaks detected in the size standard differs from that in the annotation file, DNAvi will document the discrepancy and request an updated annotation file. Marker peaks can be flagged in the size standard annotation file and are excluded from normalization by default; however, they can be included via a command-line argument. Next, intermediate positions are interpolated to assign base pair values across all positions. To account for differences in sample concentration, DNAvi performs a per-sample intensity normalization to a value between 0 and 1, based on all intensity values for the individual sample, ensuring comparability across lanes and gels. The normalization step is optional and can be omitted by specifying a command-line option.

#### 2.3.3 Peak size, nucleosomal fractions, gDNA flagging

DNAvi introduces two metrics for statistical comparison of cfDNA between the groups: peak size (dynamic) and nucleosomal fraction (static). *Peak sizes* are dynamic: for each sample, all identified cfDNA peaks are numbered by order of occurrence (0-based) and size-annotated. The peak size in base pairs for the *n*th peak of each group is compared. *Nucleosomal fractions* are static and complement the peak size. They are defined as the fraction of total cfDNA within a defined size range. The default step size was determined experimentally based on size profiles of cfDNA (measured with a TapeStation4150 device) and set to 200 bp, excluding small fragments below 100 bp (mononucleosomal: 100–200 bp, dinucleosomal: 201–400 bp, and so forth). To accommodate various biological scenarios, the default step size can be adjusted, and custom size ranges can be included through a command-line argument. For statistical comparisons, a sample-wide rather than a lane-specific background correction is applied, as state-of-the-art automated electrophoresis device outputs overcome limitations of manual gels, such as lane-specific contrast deviations. Additionally, DNAvi reports high molecular weight DNA as potential genomic DNA (gDNA) using default and user-defined thresholds.

#### 2.3.4 Metadata parsing

The optional, customizable DNAvi metadata table assigns grouping variables to each sample. This allows clinically relevant comparisons (e.g., healthy versus diseased) by invoking groupwise plotting and statistical comparisons of fragment sizes. The number and type of stratification conditions are not limited; any additional variables (e.g., age, sex, comorbidity, serum biomarker levels) can be incorporated by adding columns to the metadata file.

### 2.4 Output and visualization

DNAvi produces a variety of interpretable outputs for exploratory analysis and publication-ready reporting. The plot function generates customizable visualizations of cfDNA fragment distributions. Users can highlight groupings based on metadata and exclude fragments outside the user-defined upper and lower marker ranges from the plots (as flagged in a size standard annotation file). DNAvi complements the visualization by calculating key fragment length statistics for each sample, e.g., modal, mean, and median fragment sizes in base pairs, as well as distribution metrics, such as entropy, skewness, or short-to-long fragment ratio. When metadata is supplied, these metrics are automatically aggregated and stratified to support comparisons between groups. DNAvi generates a set of output files, including intensity matrices, annotated base pair tables, statistics, heatmaps, and other high-resolution plots.

## 3 Results

### 3.1 Validation on clinical datasets

To demonstrate DNAvi’s practical utility, we compared the cfDNA fragmentation profiles of either (i) cancer patients and healthy controls, or (ii) cancer patients at different disease stages, based on electrophoresis data from three independent studies (Suzawa *et al.* 2017, Lin *et al.* 2018, Trinidad *et al.* 2023). For example, DNAvi identified a reduction of the average short cfDNA fragment fraction (50–700 bp) from 62% in diagnosed neuroblastoma patients to 32% in relapsed neuroblastoma patients (*P *= .015), in line with previous results (Trinidad *et al.* 2023); or a lowered average tetranucleosomal cfDNA fraction (601–800 bp) of 28% in lung cancer patients compared to 44% in healthy controls (*P *= .014) (Suzawa *et al.* 2017) (Fig. S1, available as supplementary data at *Bioinformatics* online). DNAvi also identified an increased average size of the first mononucleosomal peak in lung cancer patients compared with healthy controls (156 bp versus 135 bp; *P *= .02). However, this finding may be attributable to the limited control sample size (*n *= 2) and would require additional validation, underscoring DNAvi’s utility for quickly screening DNA electropherogram data for potentially interesting group differences. DNAvi produced these easily interpretable and clinically informative statistics and visualizations through a seamless, automated pipeline that required no further downstream processing or manual intervention. These results demonstrate the potential of DNAvi for exploring noninvasive diagnostics, using only electropherograms, which are generated as a quality control anyway.

### 3.2 Technical validation

To ensure DNAvi’s capacity to accurately report fragment features, we performed serial dilutions of cfDNA and compared computed areas of DNAvi to results obtained through analysis with the TapeStation Software (version 5.1). DNAvi performed similarly to the TapeStation Software, achieving squared correlation coefficients (*R*^2^) between 0.91 and 0.99, based on an ordinary least-squares (OLS) linear regression (Fig. S2, available as supplementary data at *Bioinformatics* online). We next performed a cross-method validation of DNAvi and TapeStation Software results comparing them to Oxford Nanopore sequencing. Nucleosomal fractions computed by DNAvi correlated closely with Nanopore sequencing-derived fractions (Fig. S3, available as supplementary data at *Bioinformatics* online). We further characterized how lane normalization equalized peak heights in scenarios of different sample concentrations, for which DNAvi can perform an optional sample-specific marker band cropping (Fig. S4, available as supplementary data at *Bioinformatics* online). Finally, we validated DNAvi’s capacity to flag potential gDNA in a scenario where increasing amounts of gDNA were added to a cfDNA sample (Fig. S5, available as supplementary data at *Bioinformatics* online). DNAvi also accurately detected differences in distribution-based descriptors (e.g., entropy and skewness) across samples with multimodal fragment sizes (Fig. S6a, available as supplementary data at *Bioinformatics* online).

### 3.3 Benchmarking DNAvi in high-throughput scenarios

An additional advantage of DNAvi is its ability to process many samples without the need for manual interference. To showcase this capacity, we designed *in silico* electropherogram datasets of varying sample sizes (*N* = 24, *N* = 48, *N* = 96) and calculated DNAvi’s runtime. These analyses showed that DNAvi can produce outputs for dozens of samples in a few minutes (Table 1). For convenient exploration of multiple input files, DNAvi integrates outputs in summarizing statistics and plots (Fig. S6b, available as supplementary data at *Bioinformatics* online).

**Table 1:** DNAvi runtime for different sample sizes per module.^a^

*N*	24	48	96
Basic module & statistics (min)	1.4	2	5.4
Density plots (s)	20	42	78
Total (min)	1.8	2.7	6.7

a Benchmarking was performed on a 32 GB memory notebook with an Intel^®^ Core™ i7-10510U × 8 processor running Ubuntu 24.04.2 LTS.

### 3.4 Comparison with similar applications

In contrast to DNAvi, the majority of cfDNA fragmentomic analysis tools require sequencing. On the other hand, sequencing-free tools for DNA electrophoresis are usually designed as general-purpose band-calling tools, focused on advancing the image segmentation workflow, but lack critical functionalities tailored to studying cfDNA (Table 2, Table S1, available as supplementary data at *Bioinformatics* online). Table S1, available as supplementary data at *Bioinformatics* online, lists tools that were excluded from the major comparison in Table 2, as they were either no longer available, designed for other molecule types (RNA, protein), did not accept image input, did not output base pair annotations, or required sequencing. Among all tools so far available, key cfDNA metrics are missing or very limited. Only the commercial TapeStation Analysis Software outputs the percentage of cfDNA in a sample based on a fragment size cutoff, but solely in conjunction with purchasing designated reagents. In contrast, DNAvi computes nucleosomal fractions, allowing a more fine-grained biological interpretation of potential polynucleosomal DNA fractions, while reducing the risk of false-positive gDNA contamination reports. Other highly efficient band callers, such as AI-powered GelGenie (Aquilina *et al.* 2025), do not compute basic metrics such as the mean fragment size in base pairs, which reduces their usability for fragmentomic analyses. Furthermore, most tools rely on user input via a GUI, which prevents their integration into automated workflows, e.g., when hundreds of cfDNA samples are screened in a high-throughput manner. DNAvi’s simple command-line design allows screening of large numbers of samples and integration into existing workflows. Finally, DNAvi’s key competence is to harness user-defined metadata for stratification. This empowers users to rigorously evaluate the statistical significance of differences in cfDNA fragmentation patterns, allowing them to answer clinically relevant questions in an unbiased and reproducible manner.

**Table 2:** Comparison of sequencing-free tools for DNA fragmentation analysis.^a^

Tool	Target (metrics)	OSS	Language/implementation	Base pair annotation	Integration of metadata	Grouped statistics and visualization	Nucleosomal fractions
DNAvi	cfDNA (nucleosomal fractions, %cfDNA)	**✓**	Python/CLI	**✓**	**✓**	**✓**	**✓**
TapeStation	DNA, RNA, cfDNA (%cfDNA)	✗	GUI	**✓**	✗	✗	✗
GelGenie	DNA, RNA, protein	**✓**	Python/CLI, GUI	✗	✗	✗	✗
GelAnalyzer	DNA	✗	Java/GUI	**✓**	✗	✗	✗
ImageJ	DNA	**✓**	Java/GUI	**✓**	✗	✗	✗
PyElph	DNA	**✓**	Python/GUI	**✓**	✗	✗	✗

a *TapeStation Software* (Application Note: Agilent TapeStation Software 5.1 for the Agilent TapeStation Systems)*; GelGenie* (Aquilina *et al.* 2025)*; GelAnalyzer* (GelAnalyzer.com; http://www.gelanalyzer.com/; 7 August 2025, date last accessed)*; ImageJ* (Ziraldo *et al.* 2019)*; PyElph* (Pavel and Vasile 2012).

### 3.5 Limitations

Despite its functionalities, DNAvi remains limited to the sensitivity of the electrophoresis assay it evaluates. Therefore, DNAvi remains a pre-sequencing screening tool, and will likely not replace sequencing-based methods when more fine-grained fragmentomic metrics, such as jagged-ends or subnucleosomal oscillations, are of interest. However, *if* DNAvi identifies fractional aberrations, those are highly likely to be reflected in sequencing-based approaches, hence making DNAvi also a valuable pretest when selecting cohorts for sequencing-based downstream analysis. Evaluation of cfDNA with DNAvi in scenarios of low tumor burden, for example during very early stages of disease (multi-cancer early detection, MCED), or in early minimal residual disease (MRD), is intriguing but requires validation in larger clinical cohorts to define sensitivity limits.

On the software side, DNAvi does not provide a GUI and hence requires using the command line. However, the online documentation, including a step-by-step tutorial, provides substantial support for less-experienced users. Furthermore, this allows for DNAvi to be integrated into high-throughput processing pipelines, for which command-line applications are more suitable, as they allow a high level of automation. DNAvi saves results in user-defined directories but lacks a database interface. However, since all output is stored in standardized table formats, DNAvi is highly compatible with potential database extensions. Finally, DNAvi requires local installation. Although it relies solely on Python and several common packages, an installation-free, web-based version of DNAvi with a GUI is anticipated to enhance usability.

### 3.6 Conclusions

DNAvi is a user-friendly, free, and open-source tool for fast and efficient visualization and analysis of cfDNA fragmentation profiles from liquid biopsies, overcoming the need for sequencing for initial fragmentomic screenings. Additionally, no sensitive patient data is required for DNAvi, since native cfDNA fragment sizes do not contain identifiable information. While its major purpose is to aid clinicians and researchers in studying cfDNA profiles from blood, saliva, or other specimens, it in principle can serve to analyse any given nucleic acid (RNA, DNA), protein (Western blots with protein weight annotations in kDa), or related biomolecule blot. It provides visualization, extensive data outputs, and statistical reports tailored to cfDNA-specific features, along with an openly available source code, making it a suitable tool for integration into existing pipelines. Equipped with these intersectional functionalities, DNAvi has the potential to substantially facilitate the study of fragmentomics in clinical settings.

## Supplementary Material

btag041_Supplementary_Data

## Data Availability

Public data used in this article are available from the original publications (Suzawa *et al.* 2017, Lin *et al.* 2018, Trinidad *et al.* 2023). Additional data underlying this article are available in Zenodo under https://doi.org/10.5281/zenodo.18401705, and can be publicly accessed without a token required.
