# Supplementary material for: DNAvi: integration, statistics, and visualization of cell-free DNA fragment traces
Source: Bioinformatics. 2026 Jan 29;42(2):btag041. doi: 10.1093/bioinformatics/btag041 (PMC12904835; doi:10.1093/bioinformatics/btag041)
Supplement: btag041_Supplementary_Data [file btag041_supplementary_data.pdf]

Supplementary Data

to the publication

DNAvi: Integration, statistics, and visualization of cell-free DNA fragment traces

Anja Hess[1,2,3], Dominik Seelow[1,4*], and Helene Kretzmer[2,5*]

[1]Exploratory Diagnostic Sciences, Center of Genomic Medicine, Berlin Institute of Health at Charité Universitätsmedizin Berlin, Berlin, Germany. [2]Max Planck Institute for Molecular Genetics, Berlin, Germany. [3]Department of Biology, Chemistry and Pharmacy, Freie Universität Berlin, Berlin, Germany. [4]Institute of Medical Genetics and Human Genetics, Charité Universitätsmedizin Berlin, corporate member of Freie Universität Berlin and Humboldt-Universität zu Berlin, Berlin, Germany. [5]Digital Health Cluster, Hasso Plattner Institute for Digital Engineering, Digital Engineering Faculty, University of Potsdam, Potsdam, Germany.

*To whom correspondence should be addressed.

Contents

- Supplementary Figure 1 — Validation of DNAvi on internal and external cfDNA datasets
- Supplementary Figure 2 — Experimental validation of DNAvi with serial dilutions of cfDNA
- Supplementary Figure 3 — Experimental validation of DNAvi with Nanopore Sequencing
- Supplementary Figure 4 — Validation | characterization of DNAvi’s normalization modules
- Supplementary Figure 5 — Validation | characterization of DNAvi’s gDNA flagging module
- Supplementary Figure 6 — Validation of additional metrics and features
- Supplementary Table 1 — Extended tool comparison table
- References — Supplementary Data References

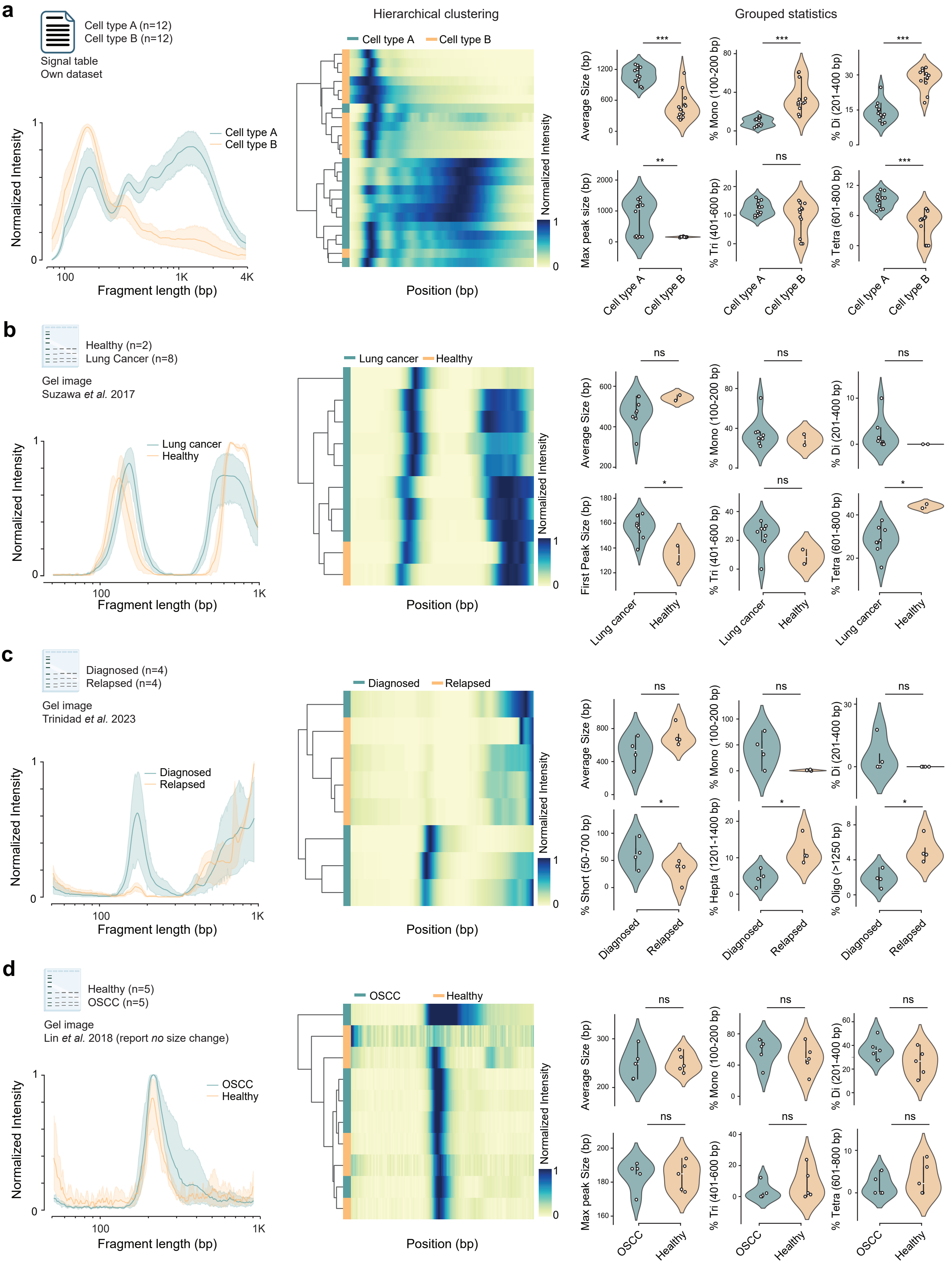

Hess, Supplementary Figure 1

Supplementary Fig. 1: Validation of DNAvi on internal and external cfDNA datasets

DNAvi was tested on in-house and published external datasets and representative outputs are shown for each vignette.
Left: Line plot showing the normalized signal intensity averaged for each group.
Center: Heatmap with hierarchical clustering (Euclidean distance) for all cfDNA samples.
Right: Violin plots showing fragment metrics in base pairs (bp) and percentage of cfDNA (%) for different nucleosomal fractions. A selection of generated violin plots focusing on identified differences between groups is shown. DNAvi displays *p*-values on top of each violin plot representing the results of the grouped comparison. *, $p < 0.05$; **, $p < 0.01$; ***, $p < 0.001$.

a. *DNAvi discriminates cfDNA from different cell types.* cfDNA was isolated from cell types A and B ($n = 12$ per group), and DNA electropherograms were generated with an Agilent Tape Station HSD5000 system, and submitted to DNAvi for analysis with per-sample marker band cropping (--correct).

b. *DNAvi identifies differences in cfDNA from healthy vs. lung cancer patients.* cfDNA gel images were obtained from (Suzawa *et al.* 2017) ($n = 2$ healthy individuals and $n = 8$ lung cancer patients), and analyzed with DNAvi.

c. *DNAvi validation on cfDNA from diagnosed vs. relapsed neuroblastoma patients.* cfDNA gel images were obtained from (Trinidad *et al.* 2023) ($n = 4$ per group), and analyzed with DNAvi running the paired mode (--paired).

d. *Control experiments confirm that DNAvi does not report statistically significant differences in cfDNA fragment lengths in oral squamous cell carcinoma (OSCC) patients, in line with the primary study.* cfDNA gel images were obtained from (Lin *et al.* 2018) ($n = 5$ per group), and analyzed with DNAvi.

a

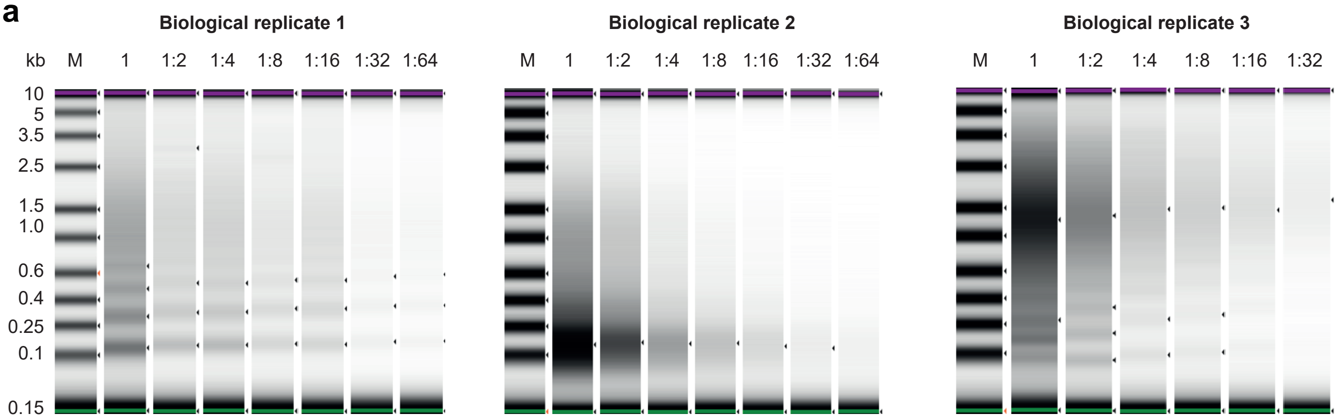

b

Dilution	Conc. [pg/µL]
undiluted	392
1:2	121
1:4	135
1:8	73.0
1:16	83.1
1:32	17.1
1:64	11.2

Dilution	Conc. [pg/µL]
undiluted	882
1:2	466
1:4	97.0
1:8	15.8
1:16	10.3
1:32	4.35
1:64	n.d.

Dilution	Conc. [pg/µL]
undiluted	1180
1:2	670
1:4	56.4
1:8	36.1
1:16	7.64
1:32	4.70

Assay quantitative range (manufacturer): 10-1000 pg/µL

c

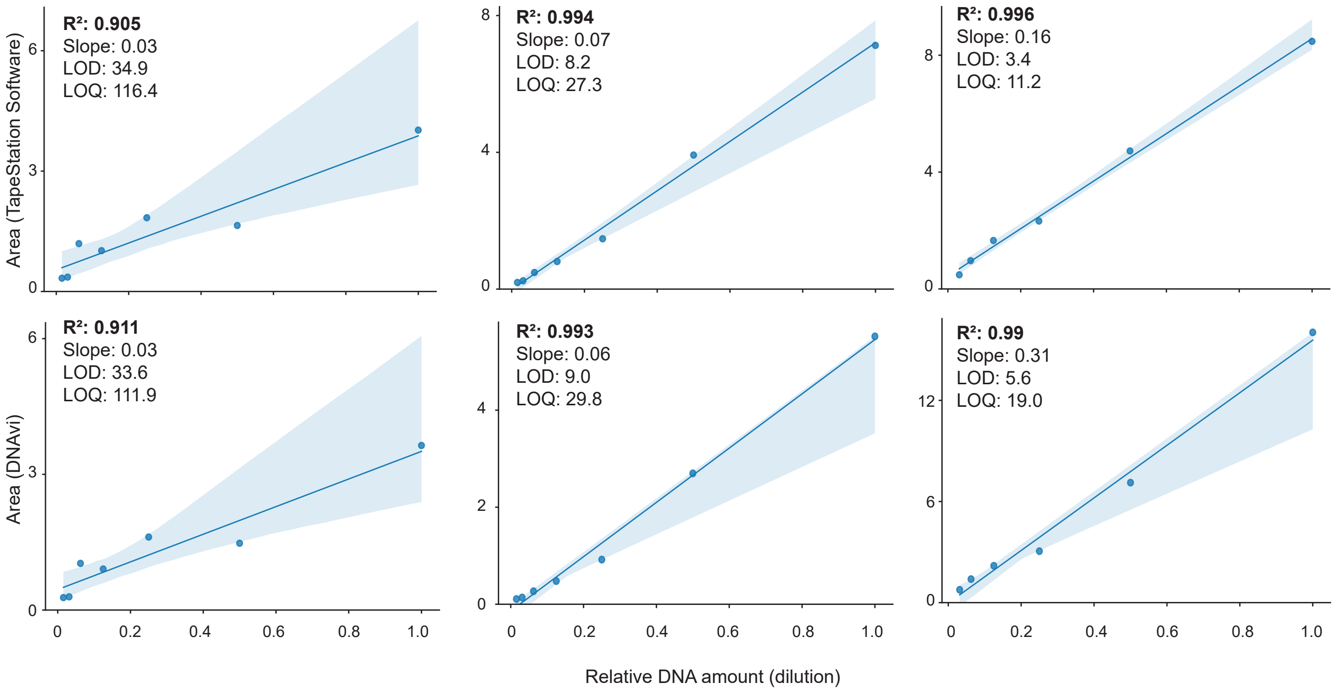

Hess, Supplementary Figure 2

Supplementary Fig. 2: Experimental validation of DNAvi with serial dilutions of cfDNA

A dilution series of cfDNA was performed in three biological replicates.

a. TapeStation gel images

b. DNA quantification (TapeStation)

c. Linear model and linearity metrics for areas measured with the TapeStation Software (top row, gold standard), and DNAvi (bottom row). Squared correlation coefficient (R^2), slope, limit of detection (LOD), and limit of quantification (LOQ) are displayed. Estimators were calculated with an ordinary least-squares (OLS) linear regression model. LOD and LOQ were estimated based on the standard deviation of the residuals. Transparent bands around the regression line are showing the 95% confidence interval.

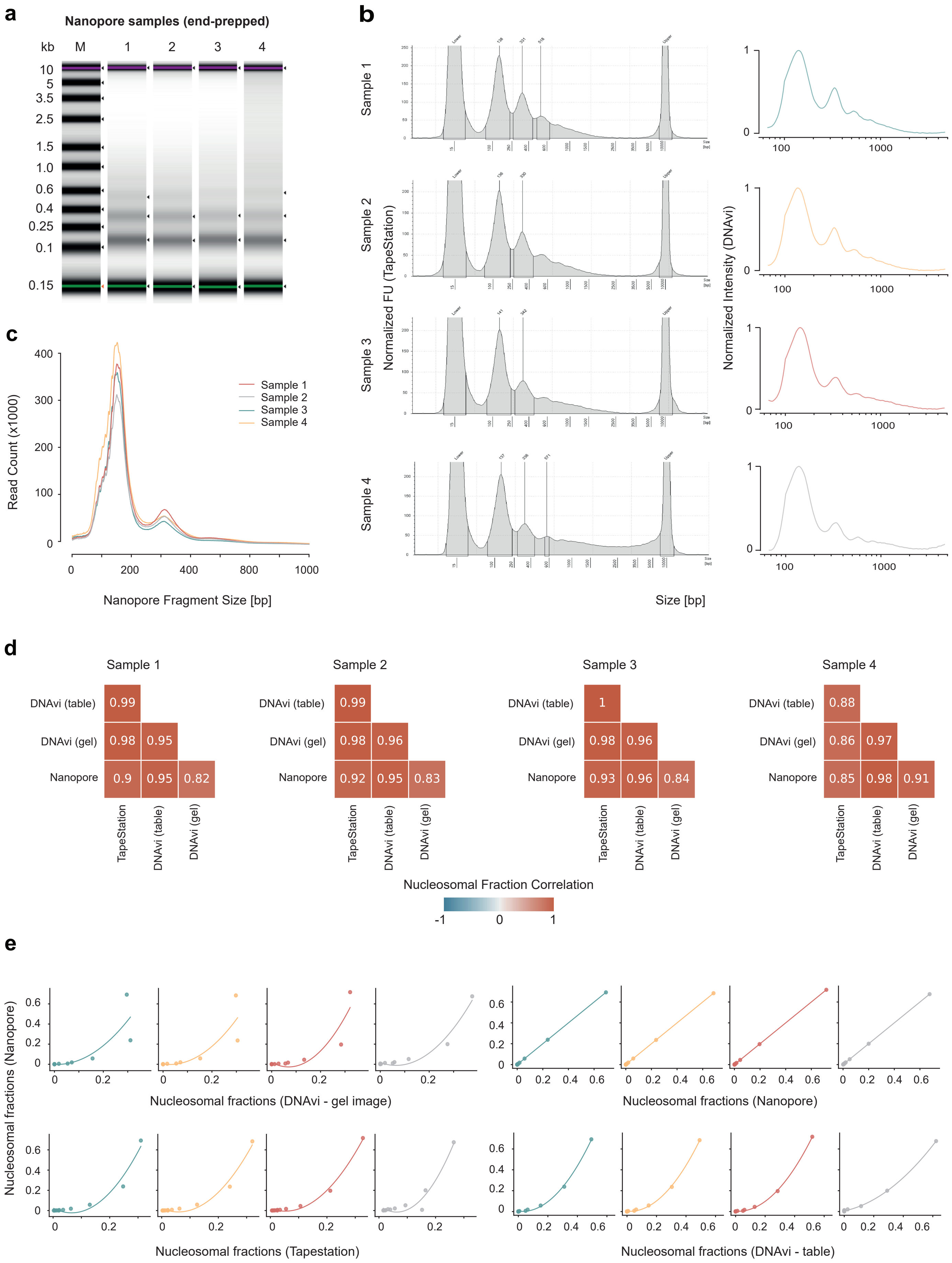

Hess, Supplementary Figure 3

Supplementary Fig. 3: Experimental validation of DNAvi with Oxford Nanopore Sequencing

a. Four cell-free DNA samples were end-repaired, bead-cleaned, and analyzed on a TapeStation device.

b. Line plots generated by TapeStation Software (left) and DNAvi (right).

c. Oxford Nanopore Sequencing-derived fragment size distributions.

d. Correlation matrices showing the Pearson correlation coefficient for nucleosomal fractions based on the different methods. Nucleosomal fractions were calculated with 3 methods: DNAvi (based on gel image or signal table), TapeStation Software, and based on Oxford Nanopore Sequencing (Native Barcoding Multiplex protocol, sequenced on a P2 flow cell).

e. Scatter plots showing the joint distribution of nucleosomal fractions for Oxford Nanopore Sequencing, TapeStation, and DNAvi outputs. Line denotes the polynomial function model fit (2. order).

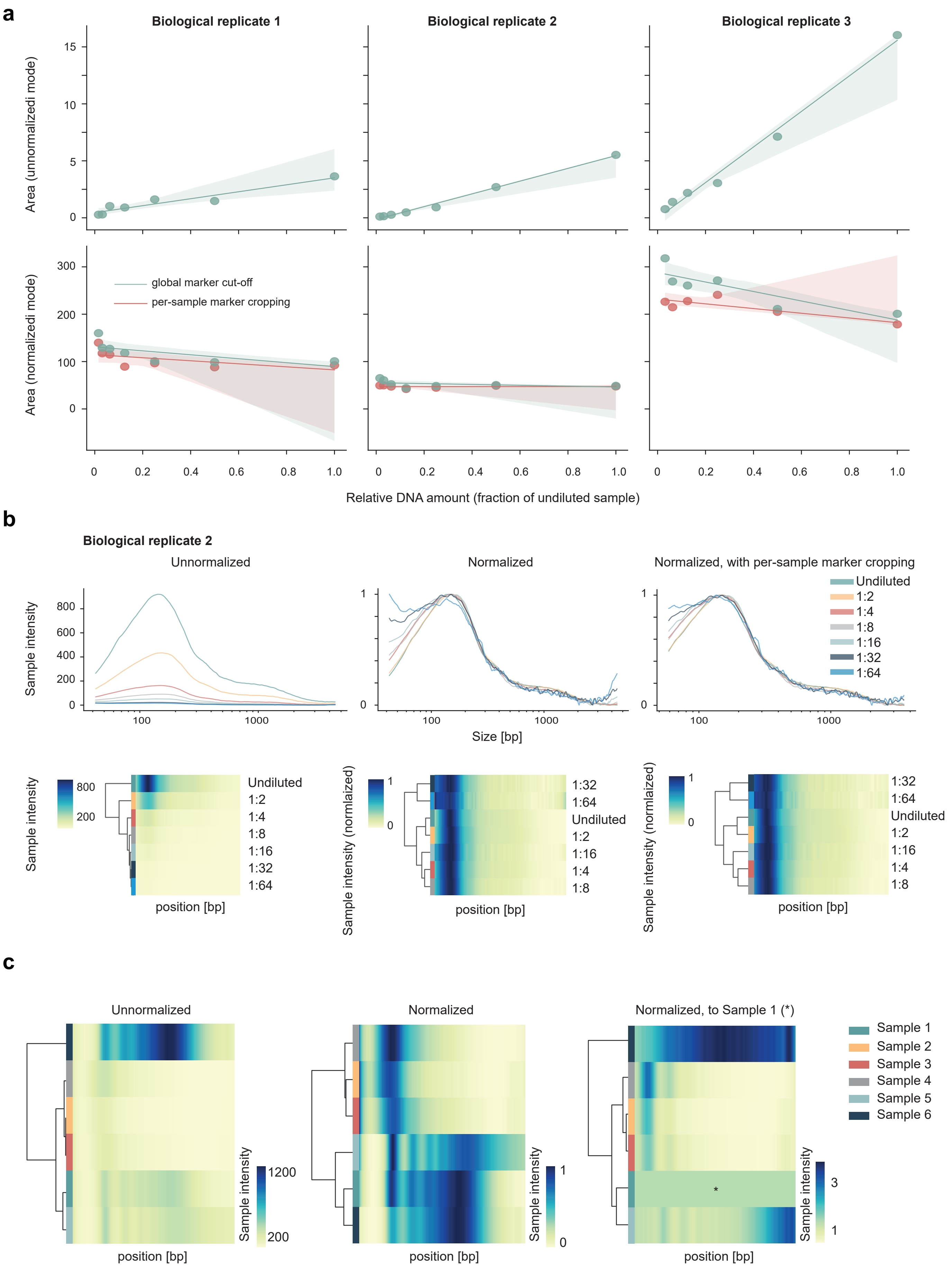

Hess, Supplementary Figure 4

Supplementary Fig. 4: Validation and characterization of DNAvi's normalization modules

a. Scatter plot showing sample area plotted against dilution for unnormalized DNAvi outputs (top row) and normalized DNAvi outputs (bottom row) for three biological replicates. Correction for high sample variability, with per-sample marker cropping, can be applied (red). Transparent bands around the regression line are showing the 95% confidence interval.

b. Detailed DNAvi output for biological replicate two. Top: Line plot for sample intensities with comparison of unnormalized DNAvi outputs (left), normalized outputs (center), and normalized outputs with per-sample marker band cropping applied (right). Note that the per-sample marker band cropping eliminates the upward line of the marker band at the lowest dilution (1:64, blue line). Bottom: Heatmaps with hierarchical clustering by Euclidean distance. Dilution strengths are annotated for each sample.

c. Example for optional per-sample normalization: Six cfDNA samples were analyzed with different DNAvi normalization modes: unnormalized (left), minimum/maximum normalized (center), and across-sample normalization to a reference sample of choice, in this case 'Sample 1' (right, the reference sample is highlighted with an asterisk).

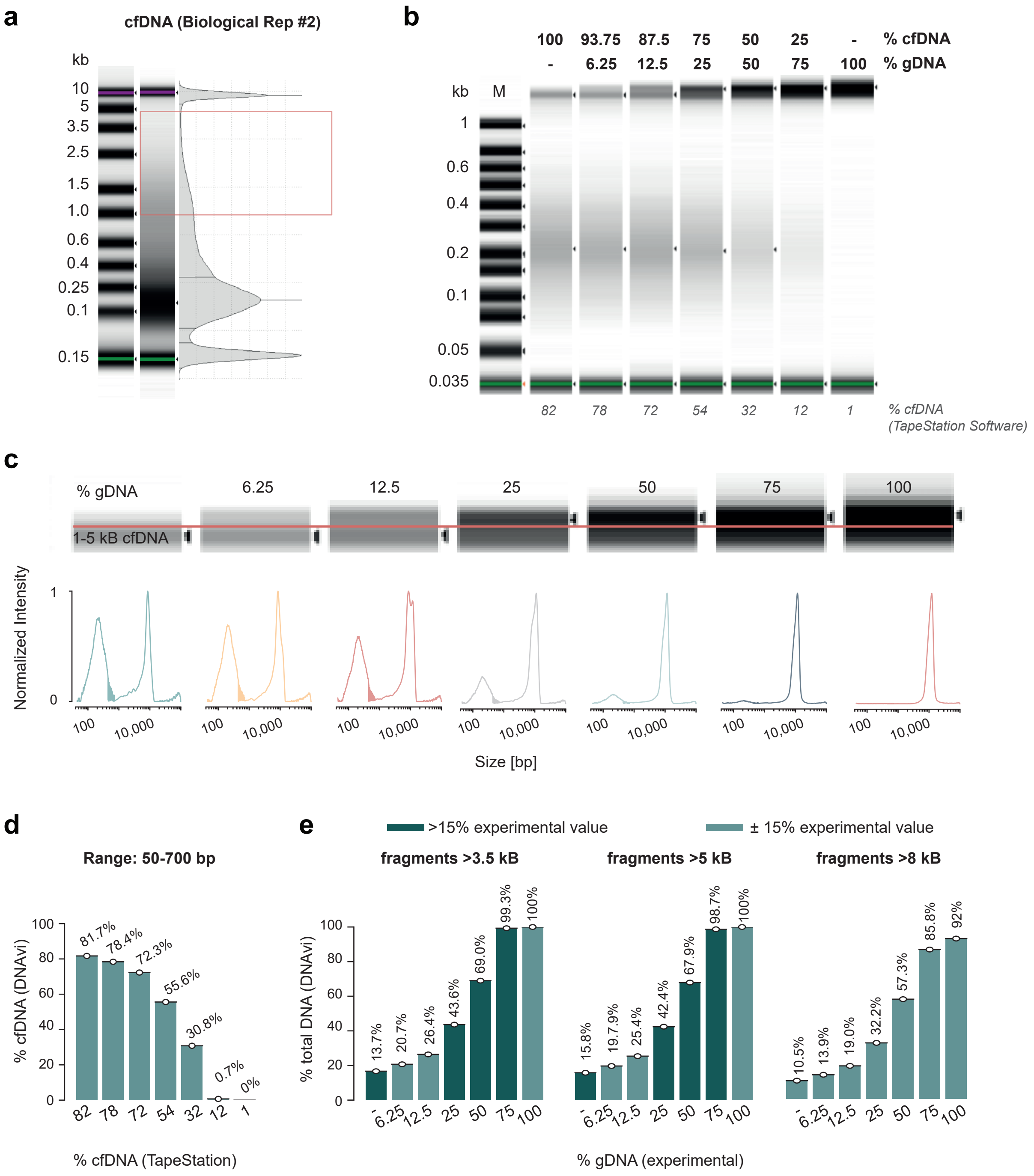
a
cfDNA (Biological Rep #2)
kb
10
5
3.5
2.5
1.5
1.0
0.6
0.4
0.25
0.1
0.15
b
100 93.75 87.5 75 50 25 - % cfDNA
- 6.25 12.5 25 50 75 100 % gDNA
kb M
1
0.6
0.4
0.2
0.1
0.05
0.035
82 78 72 54 32 12 1
% cfDNA (TapeStation Software)
c
% gDNA 6.25 12.5 25 50 75 100
1-5 kB cfDNA
Normalized Intensity
1
0
100 10,000
Size [bp]
d
Range: 50-700 bp
% cfDNA (DNAvi)
81.7% 78.4% 72.3% 55.6% 30.8% 0.7% 0%
82 78 72 54 32 12 1
% cfDNA (TapeStation)
e
>15% experimental value
± 15% experimental value
fragments >3.5 kB
fragments >5 kB
fragments >8 kB
% total DNA (DNAvi)
13.7% 20.7% 26.4% 43.6% 69.0% 99.3% 100%
15.8% 19.7.9% 25.4% 42.4% 67.9% 98.7% 100%
10.5% 13.9% 19.0% 32.2% 57.3% 85.8% 92%
- 6.25 12.5 25 50 75 100
% gDNA (experimental)

Supplementary Fig. 5: Validation and characterization of DNAvi's gDNA flagging module

a. A cfDNA sample was measured with a TapeStation HSD5000 gel to resolve fragments in the range of 1-10 kb. A fraction of the cfDNA is detected in the range of 1-5 kb (red rectangle).

b. The same sample was mixed with increasing fractions of high molecular weight gDNA, and measured with a cell-free DNA TapeStation gel (Agilent). Experimental percentages of cfDNA and gDNA are indicated on top of each lane.

c. Top: Zoomed-in display of the high-molecular weight range of the gel, red line indicates visually determined border of estimated 1-5 kb cfDNA and high molecular weight gDNA. Automatic peak annotation by the TapeStation Software displays a shift in size peak at 25 % gDNA. Bottom: DNAvi line profile plots.

d. DNAvi % cfDNA estimates for each % cfDNA value reported by the TapeStation Software (range: 50-700 bp).

e. Fractions of suspected gDNA reported by DNAvi. Dark shade: outside 15 % of the experimental value. Light shade: within 15 % of experimental value. Hyphen (x-axis) indicates the scenario where no gDNA is added, and only the ~ 1-5 kb fraction of the cfDNA can be detected.

a

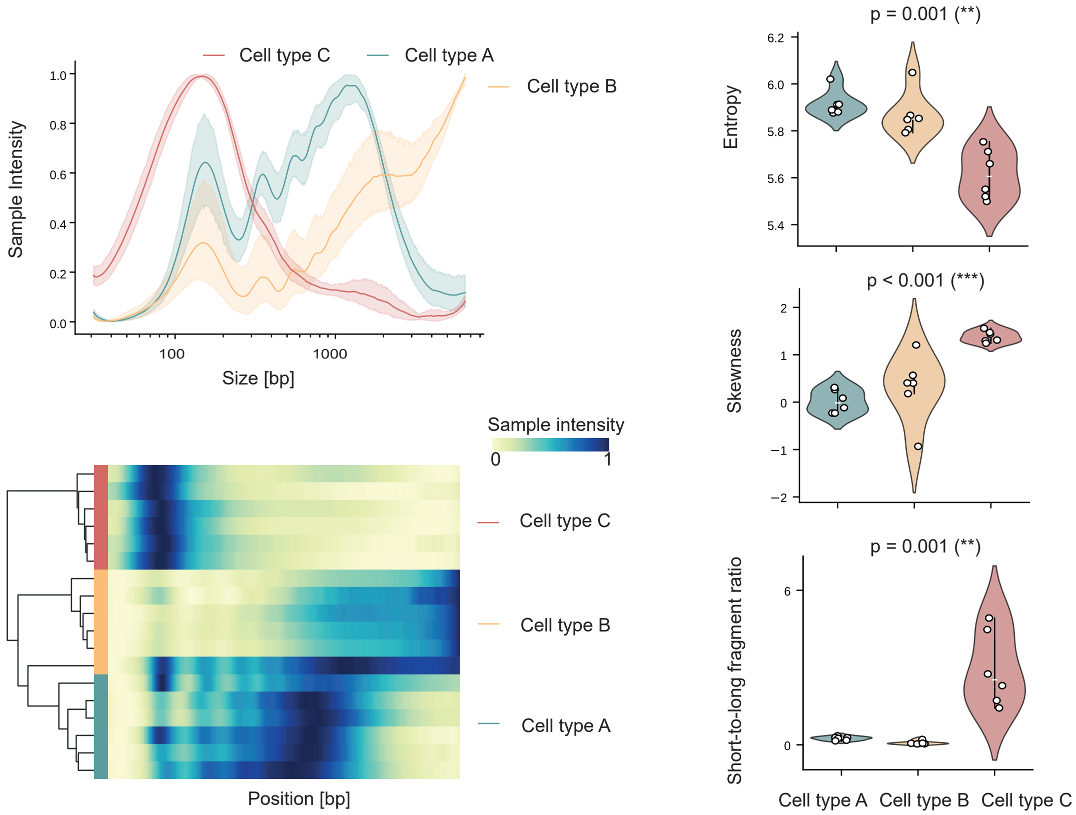
Cell type C
Cell type A
Cell type B
Sample Intensity
Size [bp]
Sample intensity
0
1
Position [bp]
p = 0.001 (**)
Entropy
p < 0.001 (***)
Skewness
p = 0.001 (**)
Short-to-long fragment ratio
Cell type A
Cell type B
Cell type C

b

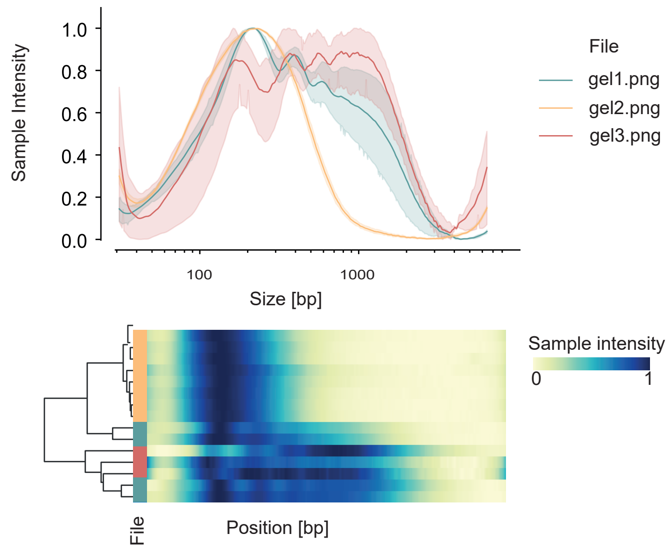
Sample Intensity
Size [bp]
File
gel1.png
gel2.png
gel3.png
Sample intensity
0
1
File
Position [bp]

Supplementary Fig. 6: Validation of additional metrics and multi-file functionalities of DNAvi

a. Example outputs for distribution metrics in DNAvi. Top-left: Line plot of cfDNA fragmentation profiles of three different cell lines. Bottom-Left: Clustered heatmap showing the DNA fluorescence intensity as a blue signal. Right: Violin plots generated from DNAvi's statistical outputs, displaying significant differences in entropy, skewness, and short-to-long fragment ratio among the three groups.

b. Examples of two DNAvi output plots for results integrated over three gel images. Top: line plot. Bottom: clustered heatmap.

Supplementary Table 1

Please note: This table was assembled based on the information available to the time of the publication. Computational tools may be subjected to change. Likewise, tools being no longer available or with limited documentation required information retrieval from publications. Therefore, minor deviations cannot be excluded. For up-to-date information on the respective tool please consult the most recent web page or repository.

**Tool (Reference)**	**Target (metrics)**	**Seq. required**	**OSS**	**Code / Download**	**Language**	**Interface**	**OS**	**Input**	**Lane/band detection**	**Base pair annotation**	**Per sample normalization**	**Advanced Metadata**	**Grouped statistics and visualization**
DNAvi	cfDNA (nucleosomal fractions, % cfDNA)	no	√	github.com/ anjahess/DNAvi	Python	CLI	Win, Unix	Image, Signal table	√	√	√	√	√
TapeStation Analysis Software (TapeStation Software Readme 5.1 2024)	(cf)DNA, RNA (% cfDNA)		✗	agilent.com	*Not publicly specified*	GUI	Win	Own format	√	√	√ (exportable as image only)	✗	✗
BioanalyzeR (Foley 2025)	DNA, RNA		√	github.com/ jwfoley/bioanalyzeR	R	Script	Win, Unix	Table	✗ *no image processing*	√	√	√ (Agilent XML file)	✗ *statistics* √*visualization*
tapeAnalyst (Fear and Qalieh)	DNA		√	github.com/jfear/ tapeAnalyst	Python	CLI	Win, Unix	Agilent Outputs	√	*not stated*	*not stated*	✗	✗
GelGenie (Aquilina *et al.* 2025)	DNA, other electrophoresis molecules		√	github.com/ mattaq31/GelGenie	Python, Java	GUI, CLI	Win, Unix	Image	√	✗	√	✗	✗
Workflow for RNA quantification (Tomlinson *et al.* 2024)	RNA		√	*enclosed to article*	ImageJ, R, Python	Script	Win, Unix	Image	✗ *full lane quantification only*	√	*to designated control band only*	✗	✗
GelAnalyzer (GelAnalyzer.com)	DNA, other electrophoresis molecules		✗	gelanalyzer.com	Java Script	GUI	Win, Unix	Image	√	√	*not stated*	✗	✗
IOCBIO Gel (Kütt *et al.* 2023)	Protein		√	gitlab.com/iocbio/gel	Python	GUI	Win, Unix	Image	√	√	*to designated control band only*	√ (staining, experiment)	✗
Gel plug-ins for ImageJ (Ziraldo *et al.* 2019)	DNA, RNA		√	imagej.net	Java/ImageJ Script	ImageJ Plugin	Win, Unix	Image	√	√	*not stated*	✗	✗
GelJ (Heras *et al.* 2015)	DNA		√	sourceforge.net/ projects/gelj	Java	GUI	Win, Unix	Image	√	√	*to designated control band only*	√	✗
PyElph (Pavel and Vasile 2012)	DNA		√	sourceforge.net/ projects/pyelph	Python	GUI	Win, Unix	Image	√	√	*not stated*	✗	✗

GelRuler (GelQuant.NET)	DNA, RNA, protein	no	✗	biochemlabsolutions.com/GelQuantNET.html	*not stated*	GUI	Win, Unix	Image	✗ *manual selection only*	✗	√ (requires manual band selection)	✗	✗
LaneRuler (Wong *et al.* 2010)	DNA		√	bcgsc.ca/resources/software/laneruler	C, Java	CLI, GUI	Unix	Image	√	✗ *external tool required*	√	✗	✗
SAFA (Simmons *et al.* 2009)	RNA		√	simtk.org/svn/safa/branches/safav11/	MATLAB	GUI	Win, Unix	Image	√	√ *nucleotide cleavage sides*	√	*not applicable* (chemical footprinting tool)	

General purpose gel analysis tools that are no longer available

Tool (Reference)	Target (metrics)	Seq. required	OSS	Code / Download	Language	Interface	OS	Input	Lane/band detection	Base pair annotation	Per sample normalization	Metadata	Grouped statistics and visualization
MatGel (Tiwari, Williams and Shan 2022)	Protein	no	✗ *code not avail-able*	*not available*	MATLAB	Script	Win, Unix	Image	√	✗	✗	✗	✗
EGBIOIMAGE (Alnamoly, Alzohairy and El-Henawy 2020)	DNA			*not available*	C#	GUI	Win	Image	√	√	*whole image adjustment only*	✗	✗
GELect for ImageJ (Intarapanich *et al.* 2015)				*not available* (prev. biotec.or.th/gi/tools/gelect)	Java	ImageJ Plugin	Win, Unix	Image	√	*not stated*	*to designated control band only*	✗	✗
GelClust (Khakabimamaghani *et al.* 2013)				*not available* (prev. bmsu.ac.ir/Services/Event/View.aspx?OId=1766)	C#	GUI	Win	Image	√	*not stated*	*not stated*	✗	✗
GelMaster (Bajla *et al.* 2005)				*not available*	MATLAB, Visual Basic	GUI	Win	Image	√	√	*not stated*	✗	✗
BandLeader (Fuhrmann *et al.* 2003)				*not available*	MATLAB	GUI	na	Image	√	√	*band normalization* (all bands equal area)	✗	✗
Image (Sulston *et al.* 1988)				*not available* (prev. sanger.ac.uk/tool/image/)	Vax FORTRAN 77	GUI	na	Image	√	√	*not stated*	✗	✗

Tool (Reference)	Target (metrics)	Seq. required	OSS	Code / Download	Language	Interface	OS	Input	Lane/band detection Base pair annotation	Data Normalization	Metadata	Grouped statistics and visualization
**cfDNA tools, sequencing-dependent**												
CfDNAPro (Wang *et al.* 2025)	cfDNA	yes	√	github.com/hw538/ cfDNAPro	R	Script	Win, Unix	BAM	*not applicable* (no image processing)	√	√ (cohort splitting by folder name)	√
CfDNAanalyzer (Zhou *et al.* 2025)				github.com/ LiymLab/ cfDNAanalyzer	Bash, Python, R	CLI, Script	Unix	BAM, BED		√	√	√ (often requires manual scripting)
FinaleToolkit (Li, Bandaru and Liu 2024)				github.com/ epifluidlab/ FinaleToolkit	Python	CLI	Win, Unix	BAM, CRAM, BED, Fragment file		√	*not stated*	*not stated*
Fragmentstein (Balázs *et al.* 2024)				github.com/uzh-dqbm-cmi/ fragmentstein	Bash, Python	CLI	Unix	BED, BEDPE, TSV		*not applicable* (conversion tool)		
cfDNA UniFlow (Röner *et al.* 2024)				github.com/ kircherlab/cfDNA-UniFlow	Python	CLI	Unix	BAM, FASTQ		√	√	√
LBFextract (Lazzeri *et al.* 2024)				github.com/Isy89/ LBF	Python	CLI	Unix	BAM, BED		√	√ (differential transcription factor analysis)	
CfdnaPattern (Chen *et al.* 2018)				github.com/ OpenGene/ CfdnaPattern	Python	CLI	Win, Unix	FASTQ		*not applicable* (classification tool)		
**cfDNA web servers and databases, sequencing-dependent**												
CfOmics (Li *et al.* 2024)	cfDNA, cfRNA, Protome, Metabolome	yes	√	***Currently offline*** (2025-11-04): cfomics.ncrnalab.org github.com/choutianxius/cfomics.git	MySQL, Python, JavaScript	Server	*not applicable*	*not applicable* (no image processing)		√	√ (pre-defined)	√
NucPosDB (Shtumpf *et al.* 2022)	cfDNA, DNA			generegulation.org/ nucposdb/	PHP	Server				✗ (curated list of tools only)		
FinaleDB (Zheng, Zhu and Liu 2021)	cfDNA			finaledb.research.chmc.org/ github.com/epifluidlab/finaledb_portal	Python, JavaScript	Server				√	√ (pre-defined)	✗

Note: *Additional proprietary tools published before 2016 were not included as they have been compared elsewhere (Khakabimamaghani et al. 2013; Heras et al. 2016).*

References

Alnamoly MH, Alzohairy AM, El-Henawy IM. EGBIOIMAGE: A Software Tool for Gel Images Analysis and Hierarchical Clustering. *IEEE Access* 2020;**8**:10768–81.

Aquilina M, Wu NJW, Kwan K *et al.* GelGenie: an AI-powered framework for gel electrophoresis image analysis. *Nat Commun* 2025;**16**:4087.

Bajla I, Holländer I, Fluch S *et al.* An alternative method for electrophoretic gel image analysis in the GelMaster software. *Computer Methods and Programs in Biomedicine* 2005;**77**:209–31.

Balázs Z, Gitchev T, Ivanković I *et al.* Fragmentstein—facilitating data reuse for cell-free DNA fragment analysis. *Bioinformatics* 2024;**40**:btae017.

Chen S, Liu M, Zhang X *et al.* A Study of Cell-Free DNA Fragmentation Pattern and Its Application in DNA Sample Type Classification. *IEEE/ACM Transactions on Computational Biology and Bioinformatics* 2018;**15**:1718–22.

Fear J, Qalieh A. *Github.Com/Jfear/tapeAnalyst*. https://github.com/jfear/tapeAnalyst/ (August 22, 2025, date last accessed)

Foley J. *Github.Com/Jwfoley/bioanalyzeR*. https://github.com/jwfoley/bioanalyzeR (August 22, 2025, date last accessed)

Fuhrmann D, Krzywinski M, Chiu R *et al.* Software for Automated Analysis of DNA Fingerprinting Gels. *Genome research* 2003;**13**:940–53.

GelAnalyzer.com. http://www.gelanalyzer.com/ (August 7, 2025, date last accessed)

GelQuant.NET. http://biochemlabsolutions.com/GelQuantNET.html (August 22, 2025, date last accessed)

Heras J, Domínguez C, Mata E *et al.* GelJ – a tool for analyzing DNA fingerprint gel images. *BMC Bioinformatics* 2015;**16**:270.

Heras J, Domínguez C, Mata E *et al.* A survey of tools for analysing DNA fingerprints. *Brief Bioinform* 2016;**17**:903–11.

Intarapanich A, Kaewkamnerd S, Shaw PJ *et al.* Automatic DNA Diagnosis for 1D Gel Electrophoresis Images using Bio-image Processing Technique. *BMC Genomics* 2015;**16**:S15.

Khakabimamaghani S, Najafi A, Ranjbar R *et al.* GelClust: A software tool for gel electrophoresis images analysis and dendrogram generation. *Computer Methods and Programs in Biomedicine* 2013;**111**:512–8.

Kütt J, Margus G, Kask L *et al.* Simple analysis of gel images with IOCBIO Gel. *BMC Biology* 2023;**21**:225.

Lazzeri I, Spiegl BG, Hasenleithner SO *et al.* LBFextract: Unveiling transcription factor dynamics from liquid biopsy data. *Computational and Structural Biotechnology Journal* 2024;**23**:3163–74.

Li JW, Bandaru R, Liu Y. FinaleToolkit: Accelerating Cell-Free DNA Fragmentation Analysis with a High-Speed Computational Toolkit. 2024:2024.05.29.596414.

Li M, Zhou T, Han M *et al.* cfOmics: a cell-free multi-Omics database for diseases. *Nucleic Acids Research* 2024;**52**:D607–21.

Lin L-H, Chang K-W, Kao S-Y *et al.* Increased Plasma Circulating Cell-Free DNA Could Be a Potential Marker for Oral Cancer. *International Journal of Molecular Sciences* 2018;**19**:3303.

Pavel AB, Vasile CI. PyElph - a software tool for gel images analysis and phylogenetics. *BMC Bioinformatics* 2012;**13**:9.

Röner S, Burkard L, Speicher MR *et al.* cfDNA UniFlow: a unified preprocessing pipeline for cell-free DNA data from liquid biopsies. *Gigascience* 2024;**13**:giae102.

Shtumpf M, Piroeva KV, Agrawal SP *et al.* NucPosDB: a database of nucleosome positioning in vivo and nucleosomics of cell-free DNA. *Chromosoma* 2022;**131**:19–28.

Simmons K, Martin JS, Shcherbakova I *et al.* Chapter 3 - Rapid Quantification and Analysis of Kinetic •OH Radical Footprinting Data Using SAFA. *Methods in Enzymology*. Vol 468. Academic Press, 2009, 47–66.

Sulston J, Mallett F, Staden R *et al.* Software for genome mapping by fingerprinting techniques. *Bioinformatics* 1988;**4**:125–32.

Suzawa K, Yamamoto H, Ohashi K *et al.* Optimal method for quantitative detection of plasma EGFR T790M mutation using droplet digital PCR system. *Oncology Reports* 2017;**37**:3100–6.

TapeStation Software Readme 5.1. https://www.agilent.com/cs/library/quickreference/public/TapeStation%20Software%20Readme_5.1.pdf (August 22, 2025, date last accessed)

Tiwari A, Williams WP, Shan X. MatGel: A MATLAB program for quantitative analysis of 2D polyacrylamide electrophoresis (2D-PAGE) protein gel images. *MethodsX* 2022;**9**:101930.

Tomlinson C, Rajasekaran A, Brochu-Gaudreau K *et al.* A convenient analytic method for gel quantification using ImageJ paired with Python or R. *PLOS ONE* 2024;**19**:e0308297.

Trinidad EM, Juan-Ribelles A, Pisano G *et al.* Evaluation of circulating tumor DNA by electropherogram analysis and methylome profiling in high-risk neuroblastomas. *Front Oncol* 2023;**13**, DOI: 10.3389/fonc.2023.1037342.

Wang H, Mennea PD, Chan YKE *et al.* A standardized framework for robust fragmentomic feature extraction from cell-free DNA sequencing data. *Genome Biology* 2025;**26**:141.

Wong RTF, Flibotte S, Corbett R *et al.* LaneRuler: Automated Lane Tracking for DNA Electrophoresis Gel Images. *IEEE Transactions on Automation Science and Engineering* 2010;**7**:706–8.

Zheng H, Zhu MS, Liu Y. FinaleDB: a browser and database of cell-free DNA fragmentation patterns. *Bioinformatics* 2021;**37**:2502–3.

Zhou J, Zhu K, Huang X *et al.* cfDNAanalyzer: a comprehensive toolkit for analyzing cell-free DNA genomic sequencing data in liquid biopsy. 2025:2025.06.09.658767.

Ziraldo R, Shoura MJ, Fire AZ *et al.* Deconvolution of nucleic-acid length distributions: a gel electrophoresis analysis tool and applications. *Nucleic Acids Res* 2019;**47**:e92.
